# Highly Conductive and Transparent AZO Films Fabricated by PLD as Source/Drain Electrodes for TFTs

**DOI:** 10.3390/ma11122480

**Published:** 2018-12-06

**Authors:** Hongke Zhang, Xiaoqing Li, Zhiqiang Fang, Rihui Yao, Xiaochen Zhang, Yuxi Deng, Xubing Lu, Hong Tao, Honglong Ning, Junbiao Peng

**Affiliations:** 1 Institute of Polymer Optoelectronic Materials and Devices, State Key Laboratory of Luminescent Materials and Devices, South China University of Technology, Guangzhou 510640, China; zhanghongkekeke@163.com (H.Z.); L_xiaoqing@163.com (X.L.); zhangxc_scut@foxmail.com (X.Z.); dengyuxixi@163.com (Y.D.); psjbpeng@scut.edu.cn (J.P.); 2 State Key Laboratory of Pulp and Paper Engineering, South China University of Technology, Guangzhou 510640, China; fangzq1230@126.com; 3 Institute for Advanced Materials and Guangdong Provincial Key Laboratory of Quantum Engineering and Quantum Materials, South China Normal University, Guangzhou 510006, China; luxubing@scnu.edu.cn; 4 Shenzhen Costar Technologies Co., Ltd., Shenzhen 518000, China; th@newvision-cn.com

**Keywords:** AZO, PLD, TFT, transparency, source/drain electrodes

## Abstract

Aluminum-doped ZnO (AZO) has huge prospects in the field of conductive electrodes, due to its low price, high transparency, and pro-environment. However, enhancing the conductivity of AZO and realizing ohmic contact between the semiconductor and AZO source/drain (S/D) electrodes without thermal annealing remains a challenge. Here, an approach called pulsed laser deposition (PLD) is reported to improve the comprehensive quality of AZO films due to the high energy of the laser and non-existence of the ion damage. The 80-nm-thick AZO S/D electrodes show remarkable optical properties (transparency: 90.43%, optical band gap: 3.42 eV), good electrical properties (resistivity: 16 × 10^−4^ Ω·cm, hall mobility: 3.47 cm^2^/V·s, carrier concentration: 9.77 × 10^20^ cm^−3^), and superior surface roughness (R_q_ = 1.15 nm with scanning area of 5 × 5 μm^2^). More significantly, their corresponding thin film transistor (TFT) with low contact resistance (R_SD_ = 0.3 MΩ) exhibits excellent performance with a saturation mobility (µ_sat_) of 8.59 cm^2^/V·s, an I_on_/I_off_ ratio of 4.13 × 10^6^, a subthreshold swing (SS) of 0.435 V/decade, as well as good stability under PBS/NBS. Furthermore, the average transparency of the unpatterned multi-films composing this transparent TFT can reach 78.5%. The fabrication of this TFT can be suitably transferred to transparent arrays or flexible substrates, which is in line with the trend of display development.

## 1. Introduction

Over the last decades, flexible and transparent electronics have attracted widespread attention. Numerous important studies on transparent materials and their application to devices have been published, especially towards the fabrication of transparent TFTs [1,2,3,4,5]. In all displays, less optical loss on transparent TFTs is unmatched. Well-designed TFTs will account for a cost reduction and life extension of displays [6,7].

Nowadays, the most commercially important material for transparent conducting material is indium tin oxide (ITO), owing to its brilliant properties of high visible transmittance (90%), low direct-current (DC) resistivity, and high infra-red reflectance [8]. Nonetheless, ITO suffers from several drawbacks, including the cost and toxicity of indium [9]. Other transparent conducting oxides (TCOs) materials are being widely developed [10,11,12]. AZO has been regarded as a promising alternative to ITO, attributed to its abundant resources, low cost, and non-toxicity [12,13,14,15,16,17]. A large number of investigators have attempted to fabricate high-quality AZO films at room temperature by various measures [18,19,20,21]. Among these ways, PLD is a relatively good method for manufacturing high-quality AZO films at room temperature, owing to the high energy of the laser and non-existence of the ion damage. Moreover, the composition of the films deposited by PLD is almost the same as that of the target.

In this paper, we try to prepare transparent TFTs by employing AZO S/D electrodes. XRD, AFM, Hall, and UV measurements are used to investigate the performances of AZO films. As a result, AZO electrodes fabricated by PLD at room temperature exhibit superior resistivity, transparency, optical band gap, and surface roughness. More significantly, the optimum AZO (PLD-AZO) S/D electrodes are used to produce transparent TFTs without thermal annealing and their corresponding devices exhibit a µ_sat_ of 8.59 cm^2^/V·s, an SS of 0.435 V/decade, an I_on_/I_off_ ratio of 4.13 × 10^6^, a high transmittance of 78.5%, and excellent stability under PBS/NBS. Furthermore, the contact resistance of TFTs is as low as 0.3 MΩ, resulting in better output characteristics.

## 2. Experimental

**Figure 1:**
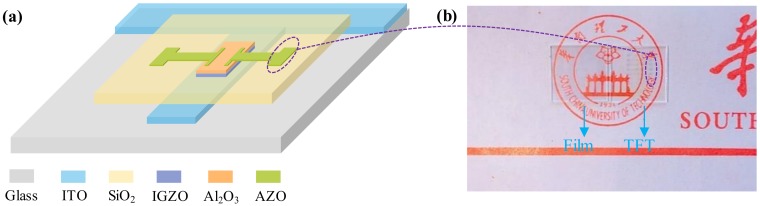
(**a**) Schematic illustration of the transparent TFTs with AZO S/D electrodes; (**b**) digital photo of transparent AZO film and corresponding TFTs.

For convenience in this paper, PVD-AZO and PLD-AZO denote the AZO electrodes prepared by PVD and PLD, respectively. PVD-AZO-TFT and PLD-AZO-TT denote their corresponding TFTs. The picture of this transparent AZO film is presented in Figure 1b. 80-nm-thick AZO films were deposited by the PVD and PLD method on the glass substrate. PVD-AZO (Al_2_O_3_: ZnO = 2:98 wt %) was fabricated by radio frequency (RF) magnetron sputtering (Kurt J. Lesker, Jefferson Hills, PA, USA) at the optimized condition (atmosphere: Pure Ar, pressure: 1 mtorr, power: 80 W). PLD-AZO (Al_2_O_3_: ZnO = 2:98 wt %) was deposited by PLD at room temperature with an O_2_ flow rate of 0 sccm, a pulsing energy of 400 mJ, a repeating rate of 5 Hz, and a KrF laser wavelength of 248 nm.

A schematic illustration and picture of the transparent TFT with AZO S/D electrodes is presented in Figure 1a,b. 200-nm-thick ITO gate electrodes and a 200-nm-thick SiO_2_ gate insulator were prepared by PVD and PECVD. Then, a bi-layer of 9.5-nm-thick indium gallium zinc oxide (IGZO) and 3.5-nm-thick Al_2_O_3_ acted as an active layer, IGZO was deposited by pulse direct-current (DC) magnetron sputtering in mixed Ar/O_2_ (100/5) atmosphere at a pressure of 1 mtorr and power of 100 W, and the pulsing frequency and reverse time for the pulse-DC mode were 10 kHz and 10 μs. Al_2_O_3_ was deposited by RF sputtering in pure Ar atmosphere, the sputtering power and pressure were 120 W and 1 mtorr [22], respectively. Finally, 80-nm-thick PVD-AZO and PLD-AZO were adopted to the S/D electrodes. The channel layer, gate insulator, S/D, and gate electrodes were all patterned by shadow masks. Additionally, S/D electrodes were fabricated at room temperature.

## 3. Results and Discussion

The excellent electrical and optical properties of AZO film enable it to be potential S/D electrodes for transparent TFTs. Among these characteristics, the resistivity, transmittance, optical band gap, contact resistance, and surface topography of AZO film are the most critical parameters for its application in transparent displays. The respective parameters of PVD-AZO and PLD-AZO that we have obtained are summarized in Table 1.

**Figure 2:**
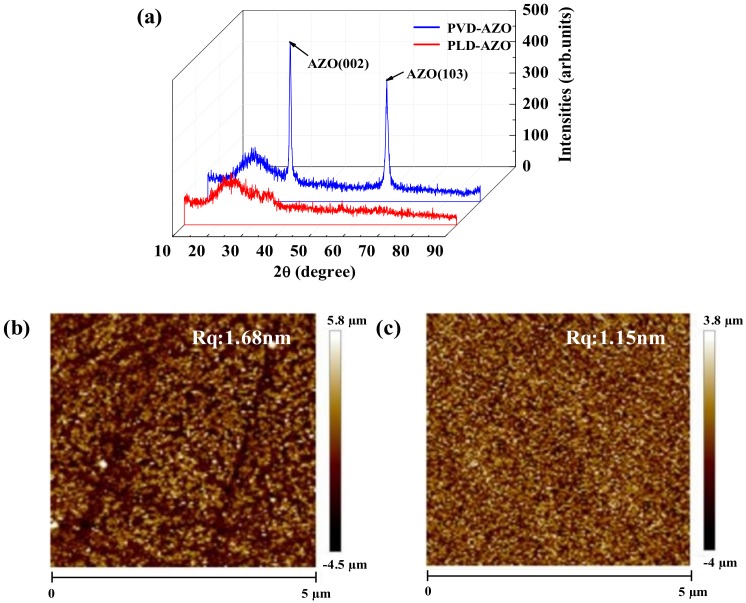
(**a**) XRD spectra of PVD-AZO and PLD-AZO; the surface topography of (**b**) PVD-AZO and (**c**) PLD-AZO.

The differences of PVD-AZO film and PLD-AZO film in performance can be associated with the scattering effect and surface topographies. Therefore, an important investigation on roughness and crystallinity of these AZO films was carried out. Figure 2a shows the XRD (PANalytical, Almelo, The Netherlands) patterns of PVD-AZO and PLD-AZO. As we can see from the XRD patterns, PVD-AZO presents two sharp peaks at (002) and (103) planes, which implies the crystalline structure of this film is not preferentially oriented to the c-axis. Grain boundary scattering may occur in PVD-AZO films [23]. On the other hand, PLD-AZO film with amorphous structure is more suitable for large-scale preparation because of its high uniformity. As shown in Figure 2b,c, the root mean square (RMS) roughness of PVD-AZO is approximately 1.68 nm, while that of PLD-AZO decreased to 1.15 nm. The high energy of the laser may cause the decrease of roughness. The ablated particles have huge kinetic energy for migrating over the surface of the substrate, which contribute to a dense and smooth PLD-AZO film. Moreover, more carrier scattering caused by the rough surface of PVD-AZO will impair the conductivity of films.

**Figure 3:**
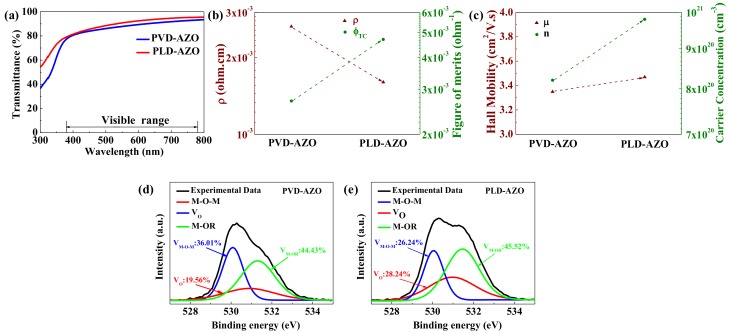
(**a**) The transmittance in the wavelength range of 300 nm–800 nm for PVD-AZO and PLD-AZO; (**b**) resistivity (ρ) and the figure of merits (Φ_TC_) of PVD-AZO and PLD-AZO; (**c**) carrier concentration (n) and Hall mobility (μ) of PVD-AZO and PLD-AZO; the O1 s region of XPS spectra for (**d**) PVD-AZO and (**e**) PLD-AZO.

As we observed from Figure 3b and Table 1, the resistivity of PLD-AZO (1.6 × 10^−3^ Ω·cm) is nearly two times smaller than that of PVD-AZO (2.64 × 10^−3^ Ω·cm), which indicates that the highly conductive PLD-AZO is more suitable for electrodes. Figure 3c shows the Hall mobility (µ) and carrier concentration (n) of PVD-AZO and PLD-AZO. The increase of µ and n of PLD-AZO may be attributed to the decrease of grain boundary scattering and the enhancement of oxygen vacancies, which can support the XRD and AFM (Multimode 8, Bruker, Billerica, MA, USA) results. The electrical properties of mental-oxide materials are mainly affected by oxygen vacancies. Figure 3d,e show the XPS (Thermo Fisher Scientific, Waltham, MA, USA) peak distribution curve of PVD-AZO and PLD-AZO. The O1 s peak can be divided into three peak components. The M-O-M peak centered at ~530 eV indicates the formation of a metal-oxygen-metal framework; the oxygen vacancies’ peak (V_O_) centered at ~531 eV is reflecting to the oxide lattice with V_O_, which is regarded as a critical factor affect the carrier concentration; the M-OR peak centered at ~532 eV is attributed to chemically adsorbed oxygen or water. The oxygen area proportion (V_O_/(V_O_ + V_M-O-M_)) denotes the relative quantity of oxygen vacancy [24]. The value of V_O_/(V_O_ + V_M-O-M_) is 0.35 for the PVD-AZO and significantly increases to 0.52 for the PLD-AZO. Thus, the XPS result shows that there are more oxygen vacancies in PLD-AZO film, corresponding to the high carrier concentration of 9.9 × 10^20^ cm^−3^. 

Another essential parameter that is measured to investigate whether the AZO films can be used in transparent display is the transmittance. Figure 3a shows the average transparency of PVD-AZO and PLD-AZO in the visible range is 88% and 90.43%, respectively. PLD-AZO is superior in both resistivity and transmittance, which means the PLD method is the better way to prepare excellent TCOs compared with PVD. The figure of merit (Φ_TC_) is also a vital aspect to evaluating the quality of transparent conductive films. As shown in Figure 2b, the higher value of Φ_TC_ (4.7 × 10^−3^ Ω^−1^) of PLD-TFT implies the better quality transparent AZO film can be deposited by PLD. Overall, the AFM, XRD, Hall, and optical results demonstrate PLD is an ideal method for fabrication of transparent AZO S/D electrodes with a dense, uniform, and smooth surface.

**Figure 4:**
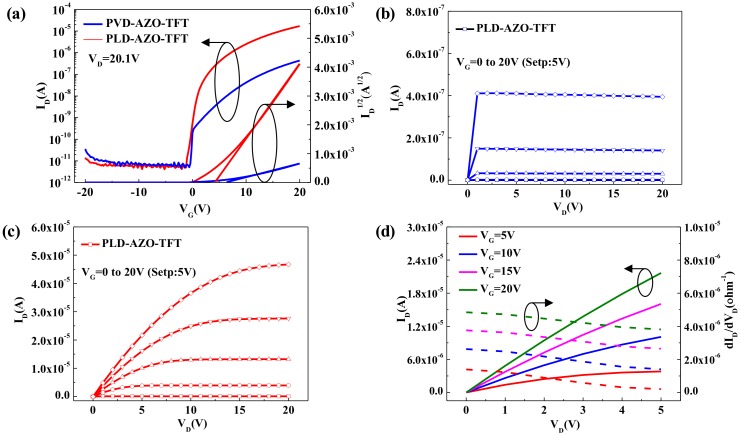
(**a**) Transfer characteristics of PVD-AZO-TFT and PLD-AZO-TFT; Output characteristics of (**b**) PVD-AZO-TFT and (**c**) PLD-AZO-TFT; (**d**) Output curve and its corresponding derivative curves in a linear region.

The bottom-gate TFTs with AZO S/D electrodes are further investigated in this paper. Figure 4 shows the transfer and output characteristics of these transparent TFTs and their corresponding respective parameters that we have measured are displayed in Table 2. As shown in Figure 4a, the PVD-AZO-TFT shows poor properties with a µ_sa_t of 0.34 cm^2^/V·s, an I_on_/I_off_ of 9.06 × 10^4^, an SS of 1.104 V/decade, and a V_th_ of 6.36 V. However, the PLD-AZO-TFT presents an excellent performance with a µ_sat_ of 8.59 cm^2^/V·s, an I_on_/I_off_ of 4.13 × 10^6^, an SS of 0.435 V/decade, and a V_th_ of 4.17 V. Figure 4b,c show the saturated output current of PVD-AZO-TFT and PLD-AZO-TFT is 0.4 μA and 46.1 μA, respectively. The PLD-AZO-TFT has better output characteristics and excellent current driving ability. As a result, the PLD method has a great effect on improving the electrical properties of TFTs. 

The S/D contact properties is a critical aspect for the output performance in a linear region [25]. To investigate the reason of the favorable performance of PLD-AZO-TFT, we researched the output curve and its corresponding derivative curves in a linear region (V_D_ = 0–5 V). Figure 4d shows the drain current linearly rose as V_D_, and the differential conductance linearly decreased as V_D_, which demonstrates the existence of high-quality ohmic contact in PLD-AZO-TFT. Additionally, the contact resistance can be obtained by the following formula of the transfer length method (TLM) [26].

**Figure 5:**
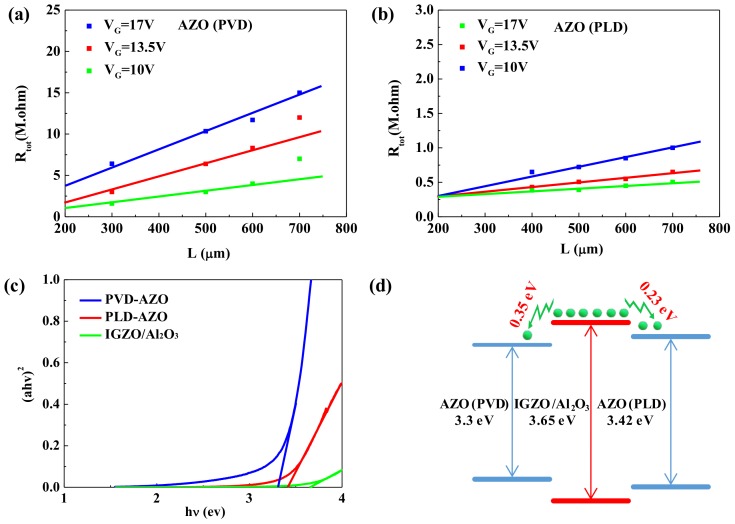
Total resistance as a function of the channel length at various V_GS_ for (**a**) PVD-AZO-TFT and (**b**) PLD-AZO-TFT; (**c**) plot of (αhν)^2^ versus hν for PVD-AZO, PLD-AZO, and IGZO/Al_2_O_3_ multi-layer; (**d**) schematic of the electron transport between AZO films and the IGZO/Al_2_O_3_ bi-layer.

R_tot_ = V_DS_/I_DS_ = L·r_ch_ + R_SD_, (1)

where R_tot_, L, r_ch_, and R_SD_ denote the total resistance, the length of the channel layer, the resistance of the channel per channel length unit, and the contact resistance, respectively. Then, R_SD_ can be obtained from the y-axis intercept by plotting R_tot_ as a function of L [27]. Figure 5a shows the R_SD_ of PVD-AZO-TFT increased from 1 MΩ to 3.8 MΩ with the increase of V_G_, which reveals there is no good ohmic contact between the IGZO/Al_2_O_3_ and PVD-AZO S/D layers. However, as shown in Figure 5b, the PLD-AZO-TFT possesses excellent performance owing to the R_SD_ keeping a constant value of 0.3 MΩ at different V_G_. The optical band gaps of AZO films and the IGZO/Al_2_O_3_ bi-layer are fitted to study the differences in the contact resistance. The E_g_ of films can be calculated by the following formula:

αhν = C(hν − E_g_)^1/2^, (2)

where α is the optical absorption coefficient, E_g_ is the band-gap energy, and hν is the photon energy [28]. The plot of (αhν)^2^ versus hν is shown in Figure 5c. The E_g_ can be obtained by fitting the straight-line portion of the plot. The value of E_g_ of PVD-AZO, PLD-AZO, and IGZO/Al_2_O_3_ multi-layer is 3.3 eV, 3.42 eV, and 3.65 eV, respectively. Based on this result, Figure 5d shows the schematic of the electron transport between AZO films and the IGZO/Al_2_O_3_ multi-layer, and the electron transport of PLD-AZO-TFT is smoother than that of PVD-AZO-TFT. This result indicates potential barriers impeding the electron transport are almost non-existent in PLD-AZO-TFT, due to a tiny difference of E_g_ between the active layer and S/D electrodes. Therefore, the R_SD_ of PLD-AZO-TFT has a relatively small value, which is related to the lower barrier. 

**Figure 6:**
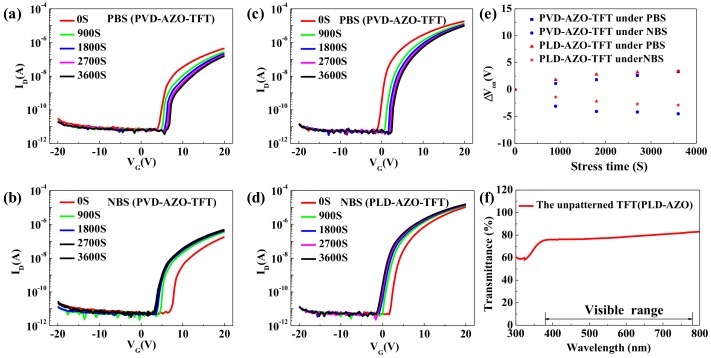
(**a**) PBS and (**b**) NBS results of the PVD-AZO-TFT; (**c**) PBS and (**d**) NBS results of the PLD-AZO-TFT; (**e**) the V_on_ shift of the TFTs under PBS (VG = +10 V) and NBS (VG = −10 V); (**f**) transmittance spectra in the wavelength range of 300 nm–800 nm for un-patterned TFTs.

The gate-bias stability of PVD-AZO-TFT and PLD-AZO-TFT were both measured and the results are displayed in Figure 6a–d. These devices were applied positive gate-bias stress (PBS) under V_GS_ = +10 V for one hour and negative gate-bias stress (NBS) under V_GS_ = −10 V for one hour. The V_on_ shift of these TFTs under the PBS and NBS measurements are compared in Figure 6e. It is evident that the V_on_ shifts of the PVD-AZO-TFT under PBS closely coincide with that of the PLD-AZO-TFT. The V_on_ shifts of the PLD-AZO-TFT under NBS are smaller than that of PVD-AZO-TFT. The NBS/PBS shifts are mainly affected by the quality of the insulator and channel layer. RF magnetron sputtering mainly relies on Ar^+^ ions’ bombard target. Few Ar^+^ ions may damage the channel layer, which brings out the relatively poor stability of PVD-AZO-TFT. Nonetheless, there is no ion damage in PLD, and the quality of the channel layer of PLD-AZO-TFT is better than that of PVD-AZO-TFT. Moreover, the ablated particles have huge kinetic energy due to the laser of PLD possessing high energy. Thus, PLD-AZO-TFT exhibit better PBS/NBS stability. 

The transparency of PLD-AZO-TFT was investigated to verify the feasibility in the transparent display. Figure 6f shows the transparency of the un-patterned PLD-AZO-TFT can reach 78.5%. Highly transparent PLD-AZO-TFT has huge potential in the field of transparent display.

## 4. Conclusions

In summary, high-performance transparent TFT with high-quality AZO source/drain electrodes was successfully fabricated by the PLD method. The resistivity, transparency, AFM, XRD, Hall, and contact resistance properties reveal different ways have great influence on dense, uniform, surface roughness, and carrier concentration of AZO films. The AZO films fabricated by the PLD method at room temperature, which possessed an exceptional quality, a resistivity of 1.6 × 10^−3^ Ω·cm, and a transmittance of 90.43%, can almost reach or even exceed that of ITO (resistivity: 6 × 10^−4^ Ω·cm; transparency: 85%) [29]. Non-toxic AZO film has great potential as a promising alternative to ITO and used in the field of environmental protection. More remarkable, TFTs with the optimized PLD-AZO electrodes exhibited excellent performance (µ_sat_: 8.59 cm^2^/V·s, I_on_/I_off_: 4.13 × 10^6^, SS: 0.435 V/decade, R_SD_: 0.3 MΩ, transparency: 78.5%), and good stability under NBS/PBS. The fabrication of this transparent TFT is desired for the transparent displays industry.

## Figures and Tables

**Table 1:** Performance parameters’ comparison of AZO films deposited by PVD and PLD.

Films	Resistivity (Ω cm)	Hall Mobility (cm^2^/V·s)	n (cm^−3^)	Transmittance	Ф_TC_ (Ω^−1^)	E_g_ (eV)
PVD-AZO	2.64 × 10^−3^	3.35	8.2 × 10^20^	88%	2.7 × 10^−3^	3.3
PLD-AZO	1.6 × 10^−3^	3.47	9.8 × 10^20^	90.43%	4.7 × 10^−3^	3.42

**Table 2:** Summary of the electrical properties of the PVD-AZO-TFT and PLD-AZO-TFT.

Devices	µ_sat_ (cm^2^/V·s)	I_on_/I_off_	SS (V/dec)	R_SD_(MΩ)	V_th_ (V)	Output Current (μA)
PVD-AZO-TFT	0.34	9.06 × 10^4^	1.104	1, 1.7, 3.8	6.36	0.4
PLD-AZO-TFT	8.59	4.13 × 10^6^	0.435	0.3	4.17	46.1
